# Periodontitis is associated to increased systemic inflammation in postmyocardial infarction patients

**DOI:** 10.1136/openhrt-2021-001674

**Published:** 2021-08-12

**Authors:** Ronaldo Lira-Junior, Elisabeth Almer Boström, Anders Gustafsson, the PAROKRANK steering committee

**Affiliations:** Department of Dental Medicine, Karolinska Institutet, Huddinge, Sweden

**Keywords:** myocardial infarction, biomarkers, inflammation

## Abstract

**Objective:**

Periodontitis has been independently associated to cardiovascular disease. However, the biological mechanisms underlying such association are still partially unknown. Thus, this study aimed to discover immunological clues accounting for the increased risk of myocardial infarction (MI) in patients having periodontitis.

**Methods:**

We included 100 patients with a first MI, 50 with and 50 without severe periodontitis, and 100 age-matched, sex-matched and area-matched controls from the Periodontitis and Its Relation to Coronary Artery Disease Study. Participants underwent comprehensive clinical and laboratory examinations 6–10 weeks after the MI and plasma expression of 92 inflammation-related markers was assessed through proximity extension assay.

**Results:**

Patients who had an MI displayed altered expression of CCL19, TNFRSF9 and LAP TGF-β1 in comparison with controls. TNFRSF9 correlated significantly with the amount of alveolar bone loss. MI patients with deep periodontal pockets showed increased white cell count and higher expression of FGF-21, HGF, OSM, CCL20 and IL-18R1 than patients without. White cell count correlated significantly with four of these proteins.

**Conclusions:**

Collectively, our results indicate molecular markers that could be responsible for the increased systemic inflammatory activity in patients with MI with periodontitis.

**Key questions**

**What is already known about this subject?**

- Periodontitis is independently associated to myocardial infarction, however, the mechanisms behind this association are still partially unknown.

**What does this study add?**

- This study reveals potential molecular markers underlying the enhanced systemic inflammatory activity associated to severe periodontitis in patients who had a myocardial infarction.

**How might this impact on clinical practice?**

- Management of periodontitis should be fostered to these patients as it may help controlling their inflammatory status.

## Introduction

Cardiovascular disease (CVD) is a major cause of premature death and chronic disability in the world, with estimates of over 420 million cases and 17 million deaths in 2015.^1^ Atherosclerosis, a central part of CVD pathophysiology, refers to the accumulation of lipids and/or fibrous material in the arteries, which can disrupt blood flow and lead to tissue ischaemia. Several established risk factors for CVD are known to activate inflammatory pathways, which can drive atherosclerosis through changes in the cells of the artery wall. Thus, inflammation itself is emerging as a risk factor for CVD,^2^ and its modulation has been shown to reduce the burden of cardiovascular illness in patients with prior myocardial infarction (MI) and ongoing subclinical inflammation.^3^

Periodontal disease comprises a variety of chronic inflammatory conditions affecting the tooth-supporting structures, which in severe cases may lead to tooth loss and negatively impact the systemic homoeostasis.^4^ Severe periodontitis has a worldwide prevalence of 11.2%, posing a huge burden on public health systems.^5^ Epidemiological studies have provided evidence that there is an independent association between periodontitis and CVD.^6 7^ Periodontitis increases systemic inflammation and pathogenic bacteria originating from the oral cavity are found in circulation as well as in atherosclerotic lesions, where they can promote vascular inflammation and atherosclerosis in animal models.^8^ However, there is still limited evidence revealing the potential biological mechanisms for which periodontitis increases the risk of CVD.

To date, studies have mainly focused on the association of periodontitis with specific markers of inflammation, such as C reactive protein (CRP), fibrinogen and interleukin (IL)-6. Nevertheless, several mechanistic pathways might explain how periodontitis is causally related to CVD.^9^ Thus, a sensitive and specific assessment of a comprehensive panel of inflammation-related markers can potentially reveal underlying biological processes linking periodontitis to CVD. Therefore, this study aimed to characterise the plasma inflammatory profile of patients who had a first MI for discovery of potential biomarkers, as well as to investigate potential pathophysiological mechanisms accounting for the increased risk of MI in patients having periodontitis.

## Methods

### Study participants

This study included participants enrolled in the multicentre, case–control Periodontitis and Its Relation to Coronary Artery Disease (PAROKRANK) Study.^6^ Briefly, PAROKRANK included patients<75 years hospitalised for a first MI at 17 Swedish hospitals (n=805). Age-matched and sex-matched controls were randomly selected from the same postal code area as the corresponding patient through the national population registry (n=805). Patients were scheduled for outpatient visits 6–10 weeks after their hospital stay at the local departments of cardiology and dental medicine, where full medical and dental examinations were performed. The matched controls were scheduled in close proximity to the outpatient visit of their corresponding patients. In the whole PAROKRANK cohort, the prevalence of severe periodontitis was 10% in the patients and 4% in the controls.^6^ For the current study, 100 MI patients, of which 50 patients were diagnosed with severe periodontitis and 50 did not have severe periodontitis, and their corresponding controls were randomly selected from the PAROKARNK database.

All participants gave their written informed consent to be a part of the study.

### Patient and public involvement

Patients were not involved in the study design.

### Clinical examination

All the participants underwent a complete physical examination including measurements of heart rate, blood pressure following 5 min of rest in a sitting position, height, body weight and waist circumference. A set of questionnaires about family and medical history, risk and health-preserving factors were also completed. Smoking was defined as current, previous (stopped >1 month ago) or never. For the patients with MI, ongoing pharmacological treatment at the time of hospital admission and follow-up visit were also recorded.

The dental examination followed a standardised protocol. Panoramic radiographs were taken from both dentate and edentulous subjects and analysed at the Department of Dental Medicine, Karolinska Institutet as described before.^6^ The radiographic examinations were performed by trained dentists blinded to whether it came from a patient with MI or a control. Participants were classified, based on the mean value of all teeth, as: healthy (≥80% remaining bone), mild to moderate periodontitis (from 79% to 66%) and severe periodontitis (<66%). Additionally, probing pocket depth (PPD) at four sites per tooth was measured.

### Plasma collection

Participants fasted 12 hours, including abstaining from smoking, before visiting the cardiology department. Venous blood was sampled according to routine procedures and the following parameters were analysed at the local laboratory: complete blood count, P-lipids (total and high-density lipoprotein cholesterol and triglycerides), apolipoprotein, P-creatinine, P-fibrinogen, P-glucose and glycohaemoglobin A1c (HbA1c). An oral glucose tolerance test (75 g glucose in 200 mL water) was performed for those without known diabetes mellitus. High-sensitivity CRP (hs-CRP) was analysed at a central laboratory (Redhot diagnostics, Södertälje, Sweden) by means of an ELISA method (MP Biomedicals, New York, New York, USA). Also, whole blood and plasma were collected and stored at –70°C in a central biobank at Karolinska Institutet.

### Protein profile analysis

A panel of 92 inflammation-related proteins was analysed in plasma using a high-throughput, multiplex immunoassay (Oink Bioscience, Uppsala, Sweden). A list of the proteins evaluated is presented in online supplemental table 1. Briefly, proximity extension assay was employed, where oligonucleotide-labelled antibodies on binding to their targets are brought in proximity, hybridise, and are extended by a DNA polymerase. The resulting sequence is then detected and quantified by quantitative real-time PCR with high specificity and high sensitivity.^10^ The data are presented in an arbitrary unit, normalised protein expression (NPX), on a log2 scale. High NPX value means high protein concentration. Internal controls were included in each run and intensity normalisation was applied. Proteins with detection above the limit in <50% of the samples were excluded from comparative analyses. For samples with NPX below detection, the specific detection limit was used. Seven samples did not pass quality control and thus were excluded from analysis.

10.1136/openhrt-2021-001674.supp1 Supplementary data

**Table 1:** Demographics and clinical characteristics of the participants

Variable	Non-MI (n=100)	MI (n=100)	P value*
Age (years)	59.6 (±8.8)	59.4 (±8.8)	–
Sex (male/female), n	76/24	76/24	–
Smoking at admission, n			
Current smoker	11	31	0.002
Ex-smoker	44	33	
Smoking at follow-up, n			
Current smoker	11	14	0.301
Ex-smoker	44	51	
Peripheral artery disease (n)	0	2	0.497
Diabetes (n)	14	21	0.366
Rheumatic disease (n)	16	14	0.692
Pulmonary disease (n)	5	16	0.012
Kidney disease (n)	3	3	–
WC (cm)	97.2 (±13.2)	99.3 (±12.5)	0.241
BMI (kg/m^2^)	26.7 (±4.7)	27.4 (±4.3)	0.286
SBP (mm Hg)	136.0 (±17.9)	130.7 (±17.2)	0.033
DBP (mm Hg)	83.6 (±11.4)	77.2 (±10.2)	<0.001
Pharmacological treatment at follow-up, n			
Renin–angiotensin inhibitors	13	73	<0.001
Aspirin	10	98	<0.001
β-blockers	11	91	<0.001
Statins	12	97	<0.001
Anti-inflammatory agents	3	1	0.621
Corticosteroids	5	1	0.212
Laboratory			
HbA1c (mmol/mol)	39.0 (±6.4)	40.7 (±8.4)	0.119
Triglycerides (mmol/L)	1.5 (±2.1)	1.3 (±0.7)	0.296
Total cholesterol (mmol/L)	5.4 (±1.1)	4.0 (±0.7)	<0.001
HDL cholesterol (mmol/L)	1.5 (±0.4)	1.3 (±0.4)	0.001
Fibrinogen (g/L)	3.0 (±0.7)	3.4 (±0.8)	0.001
Apolipoprotein B (g/L)	2.3 (±12.2)	0.8 (±0.2)	0.276
Apolipoprotein A1 (g/L)	3.5 (±17.8)	1.4 (±0.3)	0.319
hs-CRP (mg/L)	2.2 (±2.8)	2.4 (±2.5)	0.596
WCC (×10^9^/L)	5.5 (±1.4)	6.5 (±1.7)	<0.001
Periodontal parameters			
No of teeth	24.3 (±5.5)	21.1 (±7.9)	0.001
PPD≥4 mm (%)	11.1 (±12.6)	24.6 (±48.4)	<0.001
Bone loss (%)	18.1 (±8.4)	25.9 (±23.5)	<0.001

Continuous variables are presented as mean and SD and categorical variables as natural frequencies.

*P values were compared by t-test or χ2/Fisher’s exact test.

BMI, body mass index; DBP, diastolic blood pressure; HbA1c, glycohaemoglobin A1c; HDL, high-density lipoproteins; hs-CRP, high-sensitivity C reactive protein; MI, myocardial infarction; PPD, probing pocket depth; SBP, systolic blood pressure; WC, waist circumference; WCC, white cell count.

### Statistical analysis

Continuous variables are presented as mean and SD and categorical variables are presented in natural frequencies. Principal component analysis (PCA) was performed with proteins that passed the detectability cut-off to observe sample clustering. To evaluate the associations of covariates with the proteins, generalised linear models were built with each protein as the dependent variable and age, sex, smoking at follow-up, body mass index (BMI), systolic blood pressure and HbA1c as independent variables. Group comparisons were performed with t-test, χ^2^ or Fisher’s exact test, whenever appropriate. Pearson coefficient was calculated to assess the correlations between proteins and relevant clinical variables. To account for multiple testing, p values were adjusted using Benjamini and Hochberg method and considered significant when false discovery rate <0.05. Protein–protein interactions and gene ontology overrepresentation were performed with STRING in Cytoscape.^11^ Redundant terms were removed from overrepresentation analyses (redundancy cut-off of 0.5).

Lastly, proteins initially identified as differentially expressed in periodontitis were further investigated through linear regression models with periodontitis, age and smoking at follow-up as independent variables. At this stage, nominal p values <0.05 were considered statistically significant. Data analysis was performed at SPSS, V.25 (SPSS, IBM) and GraphPad Prism, V.8 (GraphPad Software, La Jolla, California, USA).

## Results

### Clinical characteristics of the study participants

This study included 100 patients who had a first MI and 100 age-matched and sex-matched controls. Demographics and clinical characteristics are described in table 1. Smoking and pulmonary diseases were more frequent among patients at admission (p=0.002 and p=0.012, respectively). The use of drugs for cardiovascular therapy, such as renin–angiotensin inhibitors, aspirin, β-blockers and statins, were more prevalent in patients at follow-up (p<0.05). This was accompanied by lower blood pressure and high-density lipoproteins and total cholesterol in patients (p<0.05). On the contrary, fibrinogen and white cell count (WCC) were elevated in patients at follow-up (p=0.001 and p<0.001, respectively). Regarding their periodontal status, patients presented with a worse periodontal condition as evidenced by less teeth, more pockets ≥4 mm and greater amount of radiographic bone loss than controls (p<0.05).

As per inclusion criteria in this study, 50 out of the patients had severe periodontitis. Demographics and clinical characteristics of patients with MI with periodontitis are described in table 2. Patients with MI with periodontitis were older, smoked more frequently and had higher diastolic blood pressure and heart rate (p<0.05). No significant difference was observed in the pharmacological treatment at follow-up between the groups. However, those with periodontitis had higher WCC (p=0.009). As expected, they also had less teeth, more pockets ≥4 mm and greater amount of radiographic bone loss (p<0.001).

**Table 2:** Demographics and clinical characteristics of patients who had a myocardial infarction with and without periodontitis

Variable	Non-periodontitis (n=50)	Periodontitis (n=50)	P value*
Age (years)	55.4 (±9.1)	63.4 (±6.4)	<0.001
Sex (male/female), n	40/10	36/14	0.349
Smoking at admission, n			
Current smoker	6	25	<0.001
Ex-smoker	11	22	
Smoking at follow-up, n			
Current smoker	2	12	<0.001
Ex-smoker	17	34	
Peripheral artery disease (n)	2	0	0.495
Hypertension (n)	16	13	0.509
Diabetes (n)	7	14	0.086
Rheumatic disease (n)	6	8	0.592
Pulmonary disease (n)	9	7	0.585
Kidney disease (n)	1	2	1.000
WC (cm)	100.1 (±11.3)	98.7 (±13.7)	0.578
BMI (kg/m^2^)	27.7 (±4.2)	27.1 (±4.5)	0.516
SBP (mmHg)	127.6 (±16.2)	133.8 (±17.7)	0.069
DBP (mmHg)	75.2 (±9.8)	79.3 (±10.3)	0.047
Heart rate	71.7 (±14.5)	80.0 (±22.0)	0.029
Pharmacological treatment at follow-up, n			
Renin–angiotensin inhibitors	36	37	0.692
Aspirin	50	48	0.495
β-blockers	47	44	0.487
Statins	48	49	0.495
Anti-inflammatory agents	0	1	1.000
Corticosteroids	0	1	1.000
Laboratory			
HbA1c (mmol/mol)	39.4 (±8.4)	41.9 (±8.2)	0.133
Triglycerides (mmol/L)	1.2 (±0.6)	1.4 (±0.8)	0.285
Total cholesterol (mmol/L)	3.9 (±0.7)	4.1 (±0.8)	0.207
HDL cholesterol (mmol/L)	1.2 (±0.4)	1.3 (±0.4)	0.439
Fibrinogen (g/L)	3.3 (±0.8)	3.6 (±0.8)	0.096
Apolipoprotein B (g/L)	0.7 (±0.2)	0.8 (±0.2)	0.243
Apolipoprotein A1 (g/L)	1.3 (±0.3)	1.4 (±0.3)	0.730
hs-CRP (mg/L)	2.2 (±2.4)	2.5 (±2.5)	0.569
WCC (×10^9^/L)	6.1 (±1.4)	7.0 (±1.9)	0.009
Periodontal parameters			
No of teeth	26.1 (±4.2)	16.1 (±7.6)	0.001
PPD ≥4 mm (%)	13.9 (±13.9)	35.5 (±26.4)	<0.001
Bone loss (%)	10.3 (±1.1)	41.6 (±9.2)	<0.001

Continuous variables are presented as mean and SD and categorical variables as natural frequencies.

*P values were compared by t-test or χ2/Fisher’s exact test.

BMI, body mass index; DBP, diastolic blood pressure; HbA1c, glycohaemoglobin A1c; HDL, high-density lipoproteins; hs-CRP, high-sensitivity C reactive protein; MI, myocardial infarction; PPD, probing pocket depth; SBP, systolic blood pressure; WC, waist circumference; WCC, white cell count.

### Biomarker assessment and association with health covariates and diabetes

**Figure 1:**
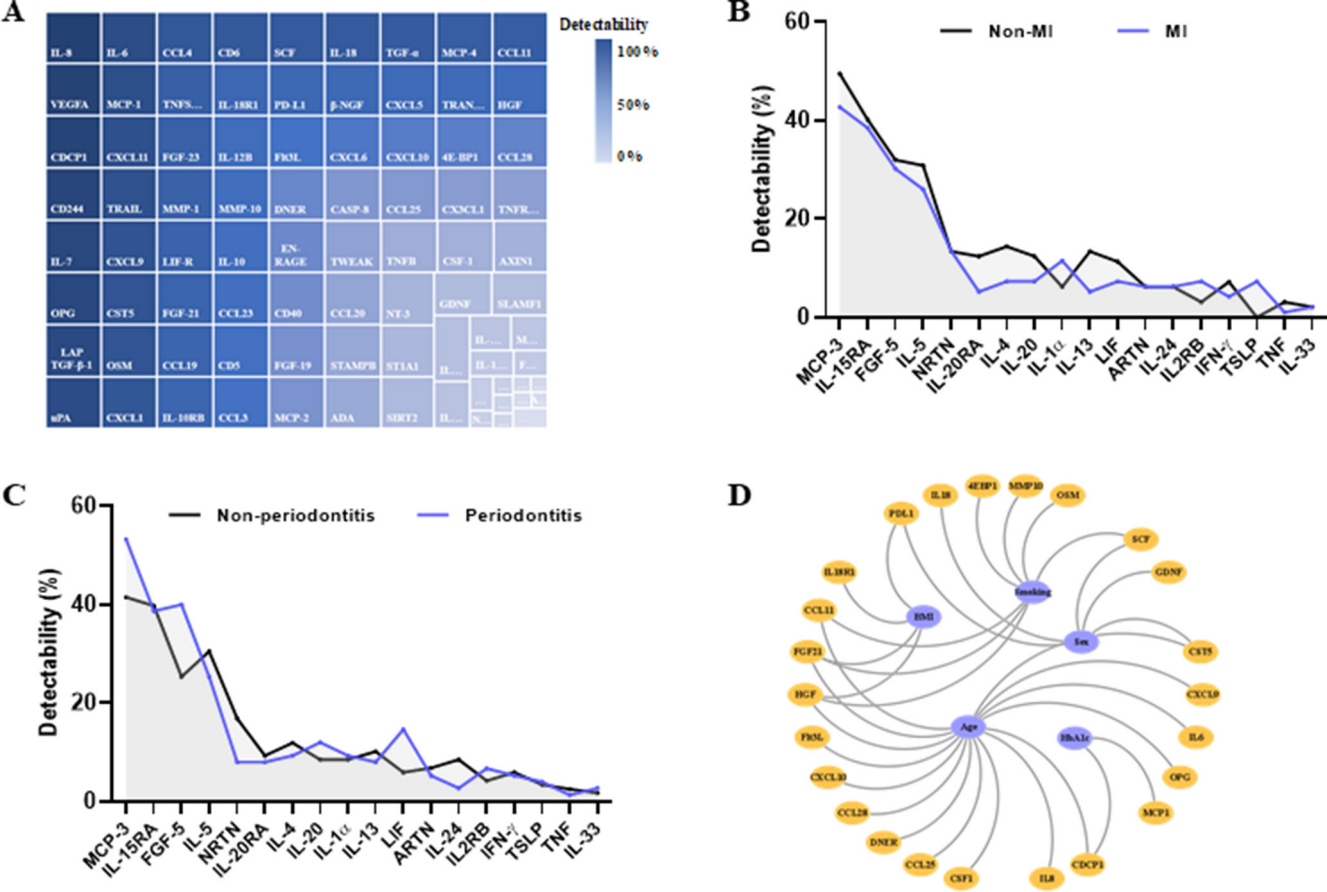
Protein detectability in plasma and their association with covariates. (A) Map of the proportions of samples with expression above detection limit for the 92 proteins assessed in plasma from the study participants. Proteins are colour coded according with the detectability. (B) Percentage of samples with expression above detection limit for the proteins with detectability <50% in myocardial infarction (MI) (blue) and non-MI (black) participants. Three proteins had all samples below detection limit and therefore are not depicted. (C) Percentage of samples with expression above detection limit for the proteins detectability <50% in periodontitis (blue) and non-periodontitis (black) participants. three proteins had all samples below detection limit and therefore are not depicted. (D) Proteins associated with the covariates age, sex, smoking, body mass index and glycohaemoglobin A1c. Only significant associations are represented (FDR<0.05). Covariates are coloured in blue and proteins in orange. FDR, false discovery rate; IL, interleukin.

The relative expression of 92 inflammation-related markers was assessed in plasma. Seven samples (3.5%) did not pass the quality control and were excluded from the analysis. Twenty-one (22.8%) markers were detected in <50% of the samples (figure 1A). Three of them were below the detection limit in all samples (BDNF, IL-2 and IL-22RA1). Then we assessed whether there were differences in the detection of the 18 analytes in participants with MI or periodontitis. Interestingly, despite the low detection frequency, TSLP was detected only in plasma from patients (7.3% vs 0%, p=0.006; figure 1B). Additionally, FGF-5 (40.0% vs 25.4%, p=0.03) and LIF (14.7% vs 5.9%, p=0.04) were detected more frequently in participants with periodontitis (figure 1C).

To evaluate the effects of important health covariates on the protein expression, we fitted generalised linear models with each protein as the dependent variable and age, sex, smoking at follow-up, BMI, systolic blood pressure and HbA1c as independent variables (figure 1D). Age had a broad effect on the protein profile, being significantly associated with 15 proteins. Sex, smoking and BMI affected 4–7 proteins each. HbA1c was significantly associated with CDCP1 and MCP-1. No protein was significantly associated with systolic blood pressure. FGF-21 and HGF were significantly associated with three covariates, age, smoking and BMI.

Further, we assessed the impact of diabetes on the protein profile. Diabetes, especially previously undetected dysglycaemia, is an important risk factor for both MI and periodontitis.^12^ We performed PCA on the 71 proteins that passed the detectability cut-off. PC1 and PC2 explained, respectively, 32.1% and 10.8% of the variance of the data. There was no evident clustering based on the presence of diabetes (online supplemental figure 1A). We also observed no clustering based on sex or smoking (online supplemental figure 2). However, participants with diabetes showed increased expression of 9 proteins, such as IL-6 and HGF (online supplemental figure 1B). These proteins displayed a high degree of protein–protein interactions (online supplemental figure 1C) and were mainly related to regulation of signalling receptor activity and monocyte chemotaxis (online supplemental figure 1D).

**Figure 2:**
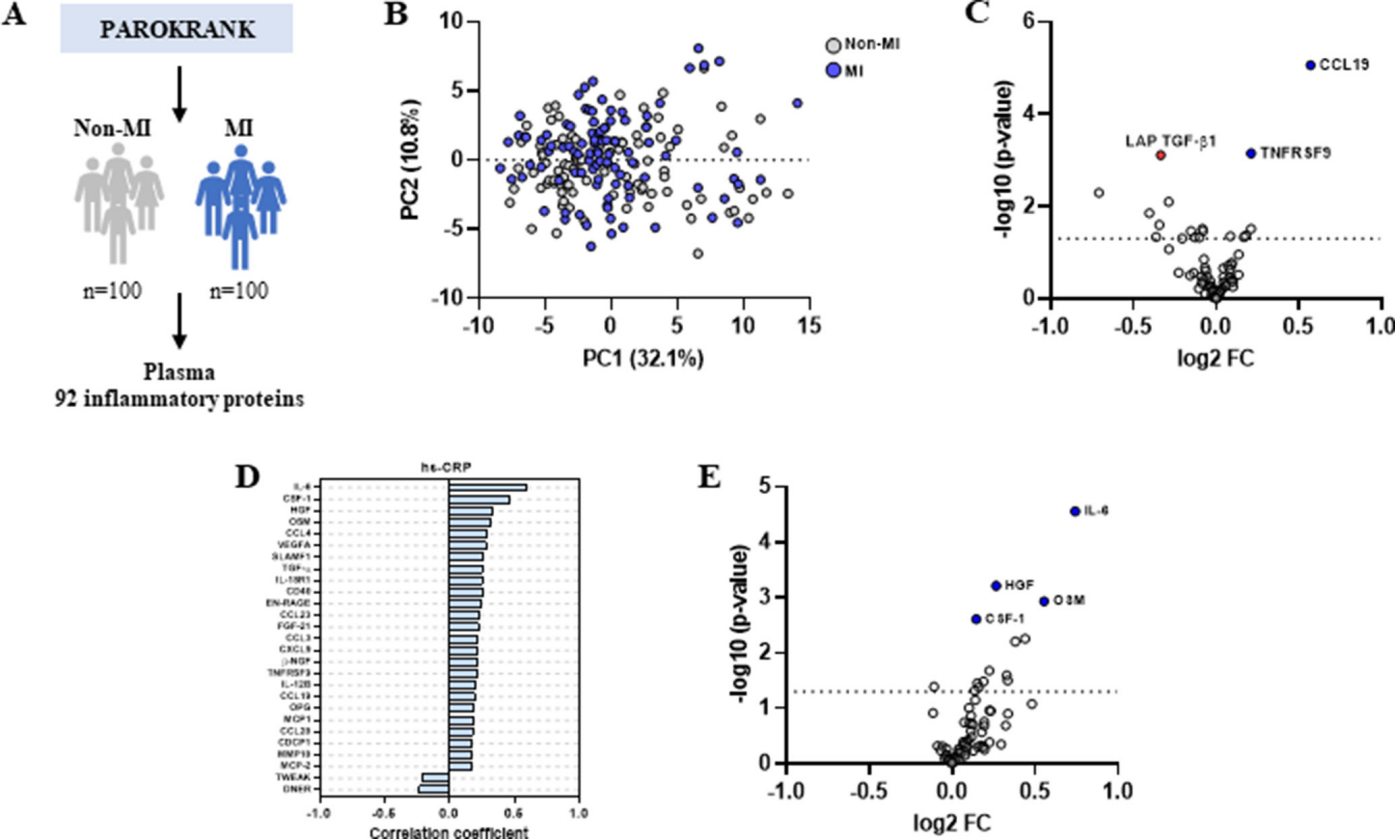
Inflammatory protein profile in plasma from patients who had a myocardial infarction (MI). (A) General outline of the study. (B) Scores plot after principal component analysis based on 71 plasma proteins showing no evident separation between MI (blue) and non-MI participants (grey). (C) Volcano plot depicting log2 fold-change (FC) in normalised protein expression and -log10 p values of plasma proteins in MI (n=96) versus non-MI controls (n=97). Significantly increased proteins in MI are shown in blue and decreased proteins in red (t-test, FDR<0.05). (D) Pearson correlations between plasma proteins and high-sensitivity C reactive protein (hs-CRP) levels. Only significant correlations (FDR<0.05) are depicted. (E) Volcano plot depicting log2 FC in normalised protein expression and -log10 p values of plasma proteins in MI patients with high hs-CRP (≥2 mg/L; n=41) versus low hs-CRP (<2 mg/L; n=55). Significantly increased proteins in patients with high hs-CRP are shown in blue (t-test, FDR<0.05). FDR, false discovery rate; PC1, principal component 1; PAROKRANK, Periodontitis and Its Relation to Coronary Artery Disease.

### Plasma protein profile in relation to MI

Next, the inflammatory protein profile in 100 MI patients and 100 matched controls was analysed (figure 2A). After a PCA analysis, no clear separation was observed between patients and controls (figure 2B). When we compared the protein expression between the groups, CCL19 and TNFRSF9 were significantly increased, whereas LAP TGF-β1 was significantly decreased in patients (figure 2C). It is interesting to note that none of these proteins altered in MI were significantly associated with any of the health covariates we assessed.

All patients with MI had been treated when the samples were collected, thus, we analysed their protein profile according to their residual inflammatory risk. We stratified the patients with MI based on their levels of hs-CRP as high risk (≥2 mg/L) or not. This cut-off has been used clinically and in trials to assess cardiovascular risk.^3 13^ hs-CRP correlated significantly with 27 proteins, two of which, TWEAK and DNER, were negative correlations (figure 2D). Furthermore, patients with high residual risk displayed significantly increased expression of IL-6, HGF, OSM and CSF-1 (figure 2E).

### Inflammatory profile of patients with MI with periodontitis

**Figure 3:**
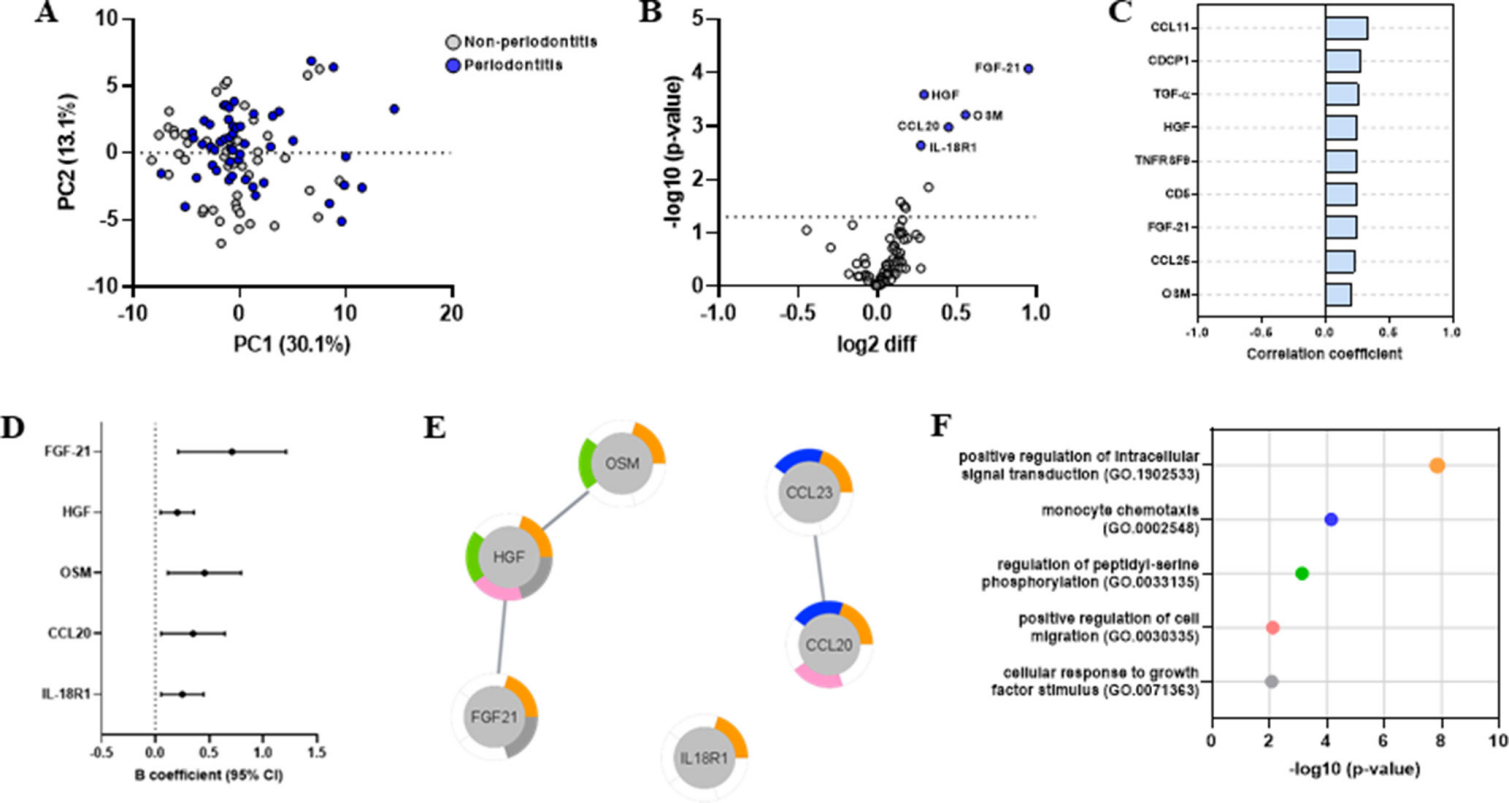
Inflammatory protein profile in plasma from periodontitis patients who had a myocardial infarction (MI). (A) Scores plot after principal component analysis based on 71 plasma proteins showing no evident separation between periodontitis (blue) and non-periodontitis participants (grey). (B) Volcano plot depicting log2 fold-change in normalised protein expression and -log10 p values of plasma proteins in MI patients with at least two pockets ≥6 mm (n=38) versus those without (n=58). Significantly increased proteins in patients with PPD ≥6 mm are shown in blue periodontitis (n=49) versus non-periodontitis patients (n=47) who had a myocardial infarction. (C) Pearson correlations between plasma proteins and radiographic bone loss in all participants. All proteins were assessed, but only significant correlations (FDR<0.05) are depicted. (D) β-coefficients and 95% CIs for the association between periodontitis and significant biomarkers identified in (B) after adjustment for age and smoking status. (E) String-based protein–protein interactions with proteins significantly altered in periodontitis. Nodes are colour coded according to their biological processes shown in (F). (F) Top five most significant gene ontology biological processes overrepresented in proteins upregulated in periodontitis. FDR, false discovery rate; IL, interleukin; PC1, principal component 1; PPD, probing pocket depth.

We set out to investigate the effects of periodontitis on the plasma protein profile from patients who had an MI. After a PCA analysis, no evident separation was seen between MI patients with and without periodontitis (figure 3A). Then, we compared the inflammatory profile of MI patients with at least two periodontal pockets (PPD) ≥6 mm to those without and found increased expression of FGF-21, HGF, OSM, CCL20 and IL-18R1 in patients with deep pockets (figure 3B). When considering the amount of bone loss, patients with MI with severe bone loss showed increased expression of nine proteins, three of which were chemokines (CCL11, CCL23 and CCL25; online supplemental figure 3A). Two of the proteins, FGF-21 and HGF, were significantly increased in both comparisons. Out of the proteins increased in periodontitis, seven correlated significantly with the amount of radiographic bone loss in all participants with correlation coefficients ranging from 0.20 to 0.34 (figure 3C). On the other hand, only OSM correlated significantly to the number of PPD≥6 mm (r=0.28; p=0.00009).

We then performed a linear regression analysis with the proteins differentially expression in periodontitis controlling for age and smoking. Both variables were significantly different between periodontitis and non-periodontitis patients (table 2) and were shown to have an impact on the plasma protein profile (figure 1D). All the proteins increased in patients with periodontal pockets ≥6 mm remained significantly associated to it after adjustment (figure 3D). On the contrary, when considering radiographic bone loss, only CCL23 remained significantly associated with periodontitis after adjustment (online supplemental figure 3B). To gain insight into the biological relevance of these findings, we show that some of the differentially expressed proteins in periodontitis, either in relation to PPD ≥6 mm or bone loss, have known protein–protein interactions (figure 3E) and are functionally related to positive regulation of intracellular signal transduction and monocyte chemotaxis, among others (figure 3F). Interestingly, both bone loss and number of PPD ≥6 mm correlated significantly with WCC (online supplemental figure 3C). WCC correlated significantly with several proteins, four of which were increased in patients with PPD ≥6 mm (online supplemental figure 3D).

## Discussion

This study investigated the influence of severe periodontitis on the plasma inflammatory profile of individuals who had a first MI. To the best of our knowledge, this is the first comprehensive evaluation of circulating inflammatory markers aiming to find inflammatory markers in plasma that could potentially contribute to a mechanistic explanation of the association between periodontitis and MI. We found that patients who had an MI displayed altered expression of CCL19, TNFRSF9 and LAP TGF-β1. The presence of deep periodontal pockets in these patients was associated with increased expression of a set of proteins mainly related to regulation of intracellular signal transduction and chemotaxis.

Patients who had an MI presented increased expression of CCL19 and TNFRSF9 and decreased expression of latent TGF-β1. Interestingly, TNFRSF9 correlated significantly with the amount of radiographic bone loss. A previous study found that serum levels of CCL19 on admission acute coronary syndrome with associated with the development of heart failure.^14^ Similarly, elevated serum levels of soluble TNFRSF9 (sCD137) in patients with acute coronary syndrome was associated with increased short-term risk of major adverse cardiovascular events.^15^ Lower frequency of LAP^+^ regulatory T cells along with lower serum TGF-β1 have been shown in patients with acute coronary syndrome.^16^ The altered expression of these proteins in stable MI patients might partially account for the increased risk of another cardiovascular event in comparison with non-MI participants.^17^ Moreover, when we consider only MI patients, those with high residual cardiovascular risk (hs-CRP ≥2 mg/L) showed increased expression of IL-6, HGF, OSM and CSF-1 in comparison to those with low residual risk. This is not surprising as the relationship between CRP and IL-6 is well known and both have been shown to be predictors of adverse cardiovascular outcome after acute coronary syndrome.^18 19^

MI participants were selected based the periodontal status, where 50 of them were diagnosed with severe periodontitis and 50 did not have severe periodontitis. This led to a very low number of participants with severe periodontitis in the control (non-MI) group. Thus, we decided to restrict our analysis to patients with MI with and without periodontitis. We explored periodontitis based on two parameters, radiographic bone loss and the presence of deep pockets. When proteins in plasma from MI patients with and without severe periodontitis were compared, nine protein were significantly higher in plasma from patients with periodontitis. However, after adjustment for age and smoking, two factors that have a strong influence on the severity of periodontitis, only one protein, CCL23, remained associated with periodontitis.

Furthermore, when assessed according to the presence of at least two periodontal pockets ≥6 mm, periodontitis was associated with increased expression of five proteins, which could be related to the elevated WCC in these patients. These five proteins, IL-18R1, FGF-21, HGF, OSM, and CCL20, differed significantly also after adjustment for age and smoking. It is not surprising that the difference, after adjustment, between patients with and without deep periodontal pockets is bigger as compared with the difference between patients with and without bone loss. While radiographic bone loss is an expression of the severity of periodontitis over time, deep pockets can be seen as an indicator of ongoing periodontal inflammation, which could have a stronger influence on the plasma proteins. Worth noting is that none of the five proteins that differed between patients with and without periodontitis differed between patients with and without MI. However, all of them correlated significantly with hs-CRP, while two of them, HGF and OSM, were increased in patients with MI with high residual risk. Most of these proteins associated with either bone loss or deep pockets have previously been related to periodontitis, such as increased concentrations of HGF and OSM in serum from periodontitis patients^20 21^ and upregulation of IL-18R1, CCL20 and CCL23 in monocytes stimulated by *Porphyromonas gingivalis* lipopolysaccharide.^22 23^ More importantly, most of them have also been associated with increased risk of CVD,^24–27^ which suggests they might be part of the pathophysiologic mechanisms explaining the increased risk of MI in periodontitis.

We also assessed the associations of important health covariates, such as age, sex, smoking, BMI, systolic BP and HbA1c, with the inflammatory proteins. We found that 24 out of 71 proteins (33.8%) were associated to at least one covariate. Age had the biggest impact on the protein profile, showing an effect on the expression of 15 proteins, which are mainly related to chemokine signalling. Sex, on the other hand, was associated with five proteins, all higher in males than in females. Age has been previously shown to influence the plasma levels of a broad range of proteins some of which were also found in the current study such as IL-8, HGF, Flt3L, OPG, CXCL9 and CXCL10.^28^ These associations might help to explain the age-associated emergence and the sex discrepancies of chronic inflammatory diseases, including CVD. Smoking had a considerable impact on the protein profile, with seven proteins associated to it. Two of the proteins identified in this study, HGF and MMP-10, have also been associated with smoking in a previous study.^29^ Interestingly, systolic BP had no significant effect on any protein. On the contrary, a previous study reported a large influence of systolic BP on biomarkers levels, many of which were also affected by age.^28^ Most of the MI participants in the current study had been treated with renin–angiotensin inhibitors and β-blockers lowering the BP, which could have influenced its association with the proteins.

We also found that diabetes was related to increased expression of nine proteins, IL-18R1, CCL11, CDCP1, OPG, HGF, TGF-α, CCL20, IL-6 and FGF-21. This is in line with a previous study reporting a major shift in the serum proteome in type 2 diabetes, partially reflecting inflammatory processes and extracellular matrix alterations.^30^ Similarly, plasma expression of HGF, IL-6, FGF-21, CDCP1 and IL-18R1 have been identified among the top 30 proteins associated with newly diagnosed type 2 diabetes.^31^ It is interesting to note that four of the proteins increased in diabetes were also increased in patients with MI deep periodontal pockets. We have previously reported in the PAROKRANK cohort that undetected diabetes was more frequent among participants with severe periodontitis.^12^ This was reflected in the sample randomly selected for the current study, where most of diabetes participants had undiagnosed disease at the time of sampling. Moreover, we believe diabetes did not play a major role in driving the differences between MI patients with and without deep periodontal pockets, as its prevalence was similar between the groups. Rather, the similarities in the altered proteins might indicate common pathways in which periodontitis and diabetes increase the risk of CVD.

This study provides limited evidence to a possible mechanistic explanation for the association between MI and periodontitis. There could be several explanations for this, such as the assessment of circulating proteins during the stable rather than acute phase of MI. Almost all patients with MI was treated with drugs having an anti-inflammatory effect such as aspirin and statins, which can be seen in the relatively low hs-CRP levels and its similar levels between patients with and without severe periodontitis. A meta-analysis has shown that patients with periodontitis have increased levels of CRP.^32^ However, we have not seen this increase in this study, which could be an effect of the medication. Another limitation of the current study could be the lack of genetic analysis as some biomarkers have a significant genetic component.^28^ Furthermore, as this was a cross-sectional study, we cannot establish any causal claim between the altered proteins, periodontitis and MI. A longitudinal study to investigate these relationships is warranted. Nevertheless, the well-characterised cohort and the unbiased assessment of a large panel of biomarkers are strengths of the study.

Altogether, we conclude that patients with MI with periodontitis have increased systemic inflammatory activity and reveal a set of proteins that might be part of the biological mechanisms linking periodontitis to CVD.

## Data Availability

Data are available on reasonable request.

## References

[1] Roth GA , Johnson C , Abajobir A , et al . Global, regional, and national burden of cardiovascular diseases for 10 causes, 1990 to 2015. J Am Coll Cardiol 2017;70:1–25. 10.1016/j.jacc.2017.04.052 28527533 PMC5491406

[2] Libby P , Buring JE , Badimon L , et al . Atherosclerosis. Nat Rev Dis Primers 2019;5:56. 10.1038/s41572-019-0106-z 31420554

[3] Everett BM , MacFadyen JG , Thuren T , et al . Inhibition of interleukin-1β and reduction in atherothrombotic cardiovascular events in the CANTOS trial. J Am Coll Cardiol 2020;76:1660–70. 10.1016/j.jacc.2020.08.011 33004131

[4] Kinane DF , Stathopoulou PG , Papapanou PN . Periodontal diseases. Nat Rev Dis Primers 2017;3:17038. 10.1038/nrdp.2017.38 28805207

[5] Kassebaum NJ , Bernabé E , Dahiya M , et al . Global burden of severe periodontitis in 1990-2010: a systematic review and meta-regression. J Dent Res 2014;93:1045–53. 10.1177/0022034514552491 25261053 PMC4293771

[6] Rydén L , Buhlin K , Ekstrand E , et al . Periodontitis increases the risk of a first myocardial infarction: a report from the PAROKRANK study. Circulation 2016;133:576–83. 10.1161/CIRCULATIONAHA.115.020324 26762521

[7] Sanz M , Marco del Castillo A , Jepsen S , et al . Periodontitis and cardiovascular diseases: consensus report. J Clin Periodontol 2020;47:268–88. 10.1111/jcpe.13189 32011025 PMC7027895

[8] Schenkein HA , Papapanou PN , Genco R , et al . Mechanisms underlying the association between periodontitis and atherosclerotic disease. Periodontol 2000 2020;83:90–106. 10.1111/prd.12304 32385879

[9] Schenkein HA , Loos BG . Inflammatory mechanisms linking periodontal diseases to cardiovascular diseases. J Clin Periodontol 2013;40 Suppl 14:S51–69. 10.1111/jcpe.12060 23627334 PMC4554326

[10] Assarsson E , Lundberg M , Holmquist G , et al . Homogenous 96-plex pea immunoassay exhibiting high sensitivity, specificity, and excellent scalability. PLoS One 2014;9:e95192. 10.1371/journal.pone.0095192 24755770 PMC3995906

[11] Doncheva NT , Morris JH , Gorodkin J , et al . Cytoscape StringApp: network analysis and visualization of proteomics data. J Proteome Res 2019;18:623–32. 10.1021/acs.jproteome.8b00702 30450911 PMC6800166

[12] Norhammar A , Kjellström B , Habib N , et al . Undetected dysglycemia is an important risk factor for two common diseases, myocardial infarction and periodontitis: a report from the PAROKRANK study. Diabetes Care 2019;42:1504–11. 10.2337/dc19-0018 31182493

[13] Goff DC , Lloyd-Jones DM , Bennett G , et al . 2013 ACC/AHA guideline on the assessment of cardiovascular risk: a report of the American College of Cardiology/American heart association Task force on practice guidelines. Circulation 2014;129:S49–73. 10.1161/01.cir.0000437741.48606.98 24222018

[14] Caidahl K , Hartford M , Ravn-Fischer A , et al . Homeostatic Chemokines and Prognosis in Patients With Acute Coronary Syndromes. J Am Coll Cardiol 2019;74:774–82. 10.1016/j.jacc.2019.06.030 31395128

[15] Yan J , Wang C , Chen R , et al . Clinical implications of elevated serum soluble CD137 levels in patients with acute coronary syndrome. Clinics 2013;68:193–8. 10.6061/clinics/2013(02)OA12 23525315 PMC3584275

[16] Zhu Z-F , Meng K , Zhong Y-C , et al . Impaired circulating CD4+LAP+ regulatory T cells in patients with acute coronary syndrome and its mechanistic study. PLoS One 2014;9:e88775. 10.1371/journal.pone.0088775 24558424 PMC3928284

[17] Haffner SM , Lehto S , Rönnemaa T , et al . Mortality from coronary heart disease in subjects with type 2 diabetes and in nondiabetic subjects with and without prior myocardial infarction. N Engl J Med Overseas Ed 1998;339:229–34. 10.1056/NEJM199807233390404 9673301

[18] Aday AW , Ridker PM . Targeting residual inflammatory risk: a shifting paradigm for atherosclerotic disease. Front Cardiovasc Med 2019;6:16. 10.3389/fcvm.2019.00016 30873416 PMC6403155

[19] Fanola CL , Morrow DA , Cannon CP , et al . Interleukin‐6 and the risk of adverse outcomes in patients after an acute coronary syndrome: observations from the SOLID‐TIMI 52 (stabilization of plaque using Darapladib—Thrombolysis in myocardial infarction 52) trial. J Am Heart Assoc 2017;6:e005637. 10.1161/JAHA.117.005637 29066436 PMC5721825

[20] Lönn J , Johansson CS , Nakka S , et al . High concentration but low activity of hepatocyte growth factor in periodontitis. J Periodontol 2014;85:113–22. 10.1902/jop.2013.130003 23594192

[21] Thorat M , AR P , Garg G . Correlation of levels of oncostatin M cytokine in crevicular fluid and serum in periodontal disease. Int J Oral Sci 2010;2:198–207. 10.4248/IJOS10077 21404969 PMC3499000

[22] Gölz L , Buerfent BC , Hofmann A , et al . Genome-wide transcriptome induced by Porphyromonas gingivalis LPS supports the notion of host-derived periodontal destruction and its association with systemic diseases. Innate Immun 2016;22:72–84. 10.1177/1753425915616685 26608307

[23] Barksby HE , Nile CJ , Jaedicke KM , et al . Differential expression of immunoregulatory genes in monocytes in response to Porphyromonas gingivalis and Escherichia coli lipopolysaccharide. Clin Exp Immunol 2009;156:479–87. 10.1111/j.1365-2249.2009.03920.x 19438601 PMC2691977

[24] Wu L , Qian L , Zhang L , et al . Fibroblast growth factor 21 is related to atherosclerosis independent of nonalcoholic fatty liver disease and predicts atherosclerotic cardiovascular events. J Am Heart Assoc 2020;9:e015226. 10.1161/JAHA.119.015226 32431189 PMC7428997

[25] Feldreich T , Nowak C , Carlsson AC , et al . The association between plasma proteomics and incident cardiovascular disease identifies MMP-12 as a promising cardiovascular risk marker in patients with chronic kidney disease. Atherosclerosis 2020;307:11–15. 10.1016/j.atherosclerosis.2020.06.013 32702535

[26] Bielinski SJ , Berardi C , Decker PA , et al . Hepatocyte growth factor demonstrates racial heterogeneity as a biomarker for coronary heart disease. Heart 2017;103:1185–93. 10.1136/heartjnl-2016-310450 28572400 PMC5511548

[27] Ganz P , Heidecker B , Hveem K , et al . Development and validation of a protein-based risk score for cardiovascular outcomes among patients with stable coronary heart disease. JAMA 2016;315:2532–41. 10.1001/jama.2016.5951 27327800

[28] Enroth S , Johansson Åsa , Enroth SB , et al . Strong effects of genetic and lifestyle factors on biomarker variation and use of personalized cutoffs. Nat Commun 2014;5:4684. 10.1038/ncomms5684 25147954 PMC4143927

[29] Huang B , Svensson P , Ärnlöv J , et al . Effects of cigarette smoking on cardiovascular-related protein profiles in two community-based cohort studies. Atherosclerosis 2016;254:52–8. 10.1016/j.atherosclerosis.2016.09.014 27684606

[30] Gudmundsdottir V , Zaghlool SB , Emilsson V , et al . Circulating protein signatures and causal candidates for type 2 diabetes. Diabetes 2020;69:1843–53. 10.2337/db19-1070 32385057 PMC7372075

[31] Gummesson A , Björnson E , Fagerberg L , et al . Longitudinal plasma protein profiling of newly diagnosed type 2 diabetes. EBioMedicine 2021;63:103147. 10.1016/j.ebiom.2020.103147 33279861 PMC7718461

[32] Paraskevas S , Huizinga JD , Loos BG . A systematic review and meta-analyses on C-reactive protein in relation to periodontitis. J Clin Periodontol 2008;35:277–90. 10.1111/j.1600-051X.2007.01173.x 18294231

