# Supplementary material for: Periodontitis is associated to increased systemic inflammation in postmyocardial infarction patients
Source: Open Heart. 2021 Aug 12;8(2):e001674. doi: 10.1136/openhrt-2021-001674 (PMC8362710; doi:10.1136/openhrt-2021-001674)
Supplement: Supplementary data [file openhrt-2021-001674supp001.pdf]

Periodontitis is associated to increased systemic inflammation in post-myocardial infarction patients

Ronaldo Lira-Junior, Elisabeth A. Boström, Anders Gustafsson; PAROKRANK steering committee*

*Steering committee acknowledgement:
Ulf de Faire – Institute of Environmental Medicine, Karolinska Institutet;
Bertil Lindahl – Department Medical Sciences, Uppsala University;
Åke Nygren – Department of Clinical Sciences Danderyd, Karolinska Institutet;
Ulf Näslund – Institution of Public Health and Clinical Medicine, Umeå University;
Per Näsman – Center for Safety Research, KTH Royal Institute of Technology;
Barbro Kjellström – Department of Medicine, Karolinska Institutet;
Kåre Buhlin – Department of Dental Medicine, Karolinska Institutet;
Elisabet Svenungsson – Department of Medicine, Karolinska Institutet;
Anna Norhammar – Department of Medicine, Karolinska Institutet;
Björn Klinge – Department of Dental Medicine, Karolinska Institutet;
Lars Rydén – Department of Medicine, Karolinska Institutet.

SUPPLEMENTAL MATERIAL

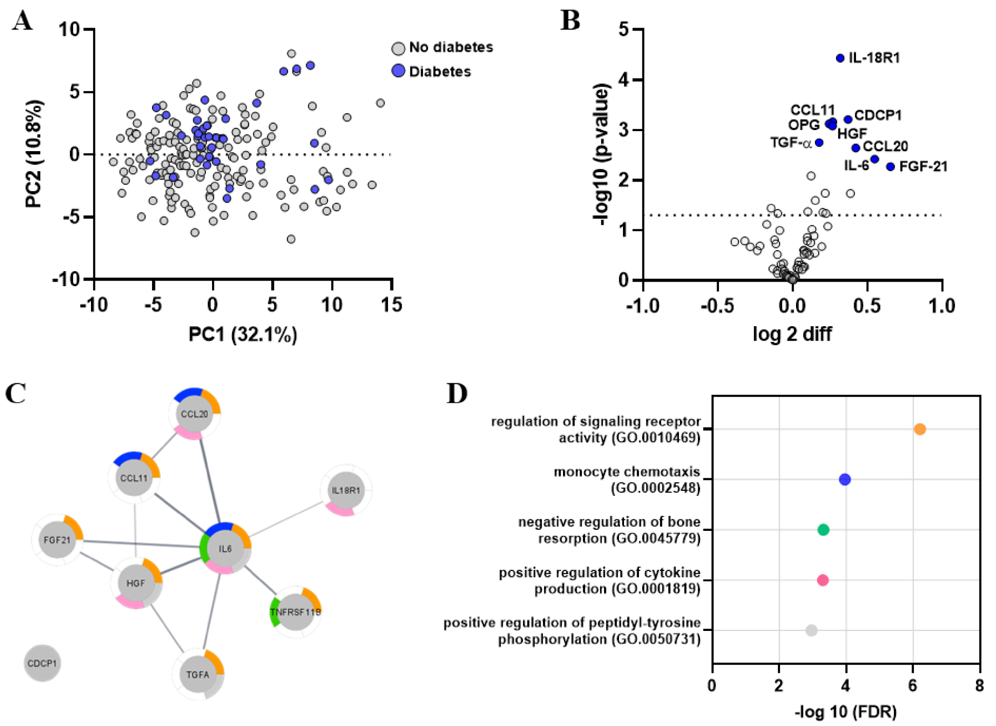

Suppl Figure 1. Influence of diabetes on the inflammatory protein profile in plasma.

A. Scores plot after principal component analysis based on 71 plasma proteins showing no evident separation between diabetes (blue) and non-diabetes participants (grey).
B. Volcano plot depicting log2 fold-change (FC) in normalized protein expression and -log10 p-values of plasma proteins in diabetes (n=35) versus non-diabetes participants (n=165) who had a myocardial infarction. Significantly increased proteins in diabetes are shown in blue (t-test, FDR<0.05).
C. String-based protein-protein interactions with proteins significantly altered in diabetes. Nodes are color-coded according to their biological processes shown in (D).
D. Top 5 most significant gene ontology (GO) biological processes overrepresented in proteins up-regulated in diabetes.

Lira-Junior R, *et al. Open Heart* 2022; 8:e001674. doi: 10.1136/openhrt-2021-001674

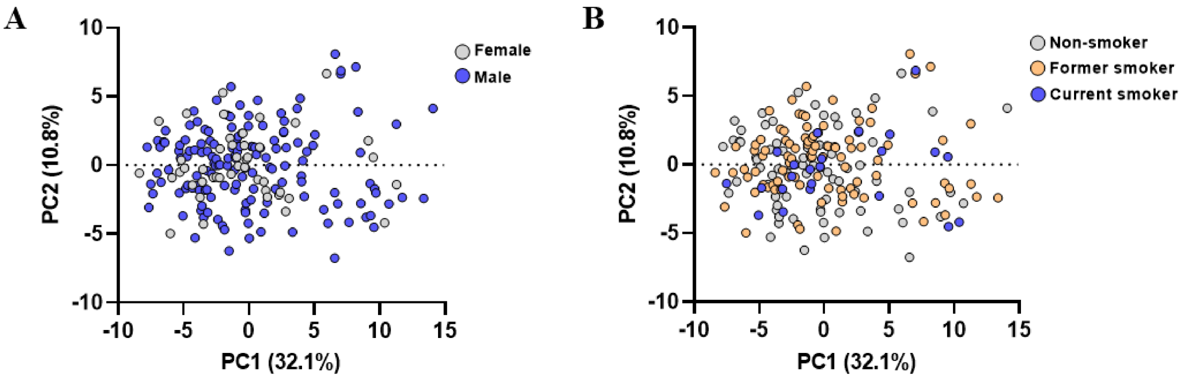

Suppl Figure 2. Plasma inflammatory profile according to sex and smoking.

A. Scores plot after principal component analysis based on 71 plasma proteins showing with samples color-coded according to sex, males (blue) and females (grey).
B. Scores plot after principal component analysis based on 71 plasma proteins showing with samples color-coded according to smoking status at follow-up visits, non-smokers (grey), former smokers (orange) and current smokers (blue).

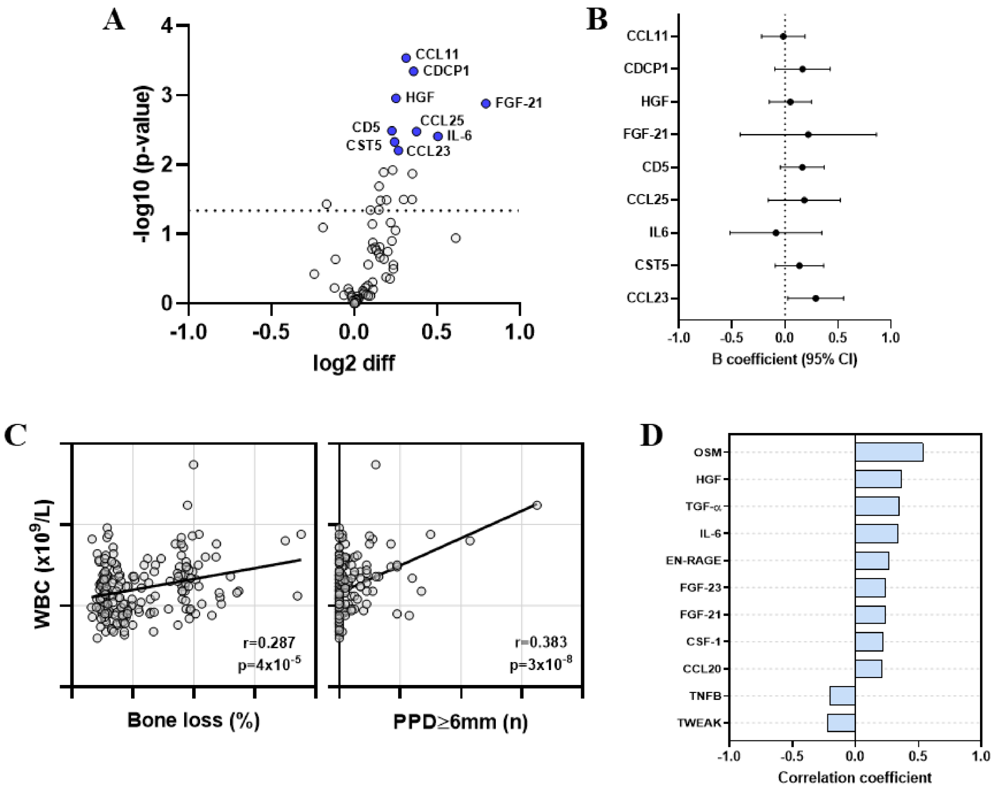

Suppl Figure 3. Inflammatory protein profile in plasma from patients who had a myocardial infarction in relation to bone loss.

A. Volcano plot depicting log2 fold-change (FC) in normalized protein expression and -log10 p-values of plasma proteins in periodontitis (n=49) versus non-periodontitis patients (n=47) who had a myocardial infarction. Significantly increased proteins in periodontitis are shown in blue (t-test, FDR<0.05).
B. β-coefficients and 95% confidence intervals for the association between periodontitis and significant biomarkers identified in (A) after adjustment for age and smoking status.
C. Pearson correlation analysis of white blood cell count (WBC) with radiographic bone loss and pocket depth (PPD) ≥ 6mm in all participants.
D. Pearson correlations between plasma proteins and WBC. All proteins were assessed, but only significant correlations (FDR<0.05) are depicted.

Suppl Table 1. List of proteins included in the Olink® Inflammation panel.

Protein	Name	Uniprot ID	Protein	Name	Uniprot ID
**4E-BP1**	Eukaryotic translation initiation factor 4E-binding protein 1	Q13541	**IL-18**	Interleukin-18	Q14116
**ADA**	Adenosine Deaminase	P00813	**IL-18R1**	Interleukin-18 receptor 1	Q13478
**ARTN**	Artemin	Q5T4W7	**IL-1α**	Interleukin-1 alpha	P01583
**AXIN-1**	Axin-1	O15169	**IL-2**	Interleukin-2	P60568
**BDNF**	Brain-derived neurotrophic factor	P23560	**IL-20**	Interleukin-20	Q9NYY1
**CASP-8**	Caspase-8	Q14790	**IL-20RA**	Interleukin-20 receptor subunit alpha	Q9UHF4
**CCL11**	C-C motif chemokine 11	P51671	**IL-22 RA1**	Interleukin-22 receptor subunit alpha-1	Q8N6P7
**CCL19**	C-C motif chemokine 19	Q99731	**IL-24**	Interleukin-24	Q13007
**CCL20**	C-C motif chemokine 20	P78556	**IL-2RB**	Interleukin-2 receptor subunit beta	P14784
**CCL23**	C-C motif chemokine 23	P55773	**IL-33**	Interleukin-33	O95760
**CCL25**	C-C motif chemokine 25	O15444	**IL-4**	Interleukin-4	P05112
**CCL28**	C-C motif chemokine 28	Q9NRJ3	**IL-5**	Interleukin-5	P05113
**CCL3**	C-C motif chemokine 3	P10147	**IL-6**	Interleukin-6	P05231
**CCL4**	C-C motif chemokine 4	P13236	**IL-7**	Interleukin-7	P13232
**CD244**	Natural killer cell receptor 2B4	Q9BZW8	**IL-8**	Interleukin-8	P10145
**CD40**	CD40L receptor	P25942	**LAP TGF-β-1**	Latency-associated peptide transforming growth factor beta-1	P01137
**CD5**	T-cell surface glycoprotein CD5	P06127	**LIF**	Leukemia inhibitory factor	P15018
**CD6**	T cell surface glycoprotein CD6 isoform	Q8WWJ7	**LIF-R**	Leukemia inhibitory factor receptor	P42702
**CDCP1**	CUB domain-containing protein 1	Q9H5V8	**MCP-1**	Monocyte chemotactic protein 1	P13500
**CSF-1**	Macrophage colony-stimulating factor 1	P09603	**MCP-2**	Monocyte chemotactic protein 2	P80075
**CST5**	Cystatin D	P28325	**MCP-3**	Monocyte chemotactic protein 3	P80098
**CX3CL1**	Fractalkine	P78423	**MCP-4**	Monocyte chemotactic protein 4	Q99616
**CXCL1**	C-X-C motif chemokine 1	P09341	**MMP-1**	Matrix metalloproteinase 1	P03956
**CXCL10**	C-X-C motif chemokine 10	P02778	**MMP-10**	Matrix metalloproteinase 10	P09238
**CXCL11**	C-X-C motif chemokine 11	O14625	**NRTN**	Neurturin	Q99748
**CXCL5**	C-X-C motif chemokine 5	P42830	**NT-3**	Neurotrophin-3	P20783
**CXCL6**	C-X-C motif chemokine 6	P80162	**OPG**	Osteoprotegerin	O00300
**CXCL9**	C-X-C motif chemokine 9	Q07325	**OSM**	Oncostatin-M	P13725
**DNER**	Delta and Notch-like epidermal growth factor-related receptor	Q8NFT8	**PD-L1**	Programmed cell death 1 ligand 1	Q9NZQ7
**EN-RAGE**	Protein S100A12	P80511	**SCF**	Stem cell factor	P21583
**FGF-19**	Fibroblast growth factor 19	O95750	**SIRT2**	SIR2-like protein 2	Q8IXJ6
**FGF-21**	Fibroblast growth factor 21	Q9NSA1	**SLAMF1**	Signaling lymphocytic activation molecule	Q13291
**FGF-23**	Fibroblast growth factor 23	Q9GZV9	**ST1A1**	Sulfotransferase 1A1	P50225
**FGF-5**	Fibroblast growth factor 5	Q8NF90	**STAMPB**	STAM-binding protein	O95630
**Flt3L**	Fms-related tyrosine kinase 3 ligand	P49771	**TGF-α**	Transforming growth factor alpha	P01135
**GDNF**	Glial cell line-derived neurotrophic factor	P39905	**TNF**	Tumor necrosis factor	P01375
**HGF**	Hepatocyte growth factor	P14210	**TNFB**	TNF-beta	P01374
**IFN-γ**	Interferon gamma	P01579	**TNFRSF9**	Tumor necrosis factor receptor superfamily member 9	Q07011
**IL-10**	Interleukin-10	P22301	**TNFSF14**	Tumor necrosis factor ligand superfamily member 14	O43557
**IL-10RA**	Interleukin-10 receptor subunit alpha	Q13651	**TRAIL**	TNF-related apoptosis-inducing ligand	P50591
**IL-10RB**	Interleukin-10 receptor subunit beta	Q08334	**TRANCE**	TNF-related activation-induced cytokine	O14788
**IL-12B**	Interleukin-12 subunit beta	P29460	**TSLP**	Thymic stromal lymphopoietin	Q969D9
**IL-13**	Interleukin-13	P35225	**TWEAK**	Tumor necrosis factor (Ligand) superfamily, member 12	O43508
**IL-15RA**	Interleukin-15 receptor subunit alpha	Q13261	**uPA**	Urokinase-type plasminogen activator	P00749
**IL-17A**	Interleukin-17A	Q16552	**VEGFA**	Vascular endothelial growth factor A	P15692
**IL-17C**	Interleukin-17C	Q9P0M4	**β-NGF**	Beta-nerve growth factor	P01138
